# Rapid and Reversible Capture of PFOS from Complex Water Matrices by an Earth-Abundant Iron(III)–Carboxylate Metal–Organic Framework

**DOI:** 10.3390/polym18172171

**Published:** 2026-09-05

**Authors:** Haoming Yang, Yuan Yu

**Affiliations:** School of Safety Science and Engineering, Nanjing Tech University, Nanjing 211816, China; yanghaoming@njtech.edu.cn

**Keywords:** PFOS, metal–organic framework, adsorption, complex water matrices, reversibility, PFAS remediation

## Abstract

Background: Perfluorooctane sulfonate (PFOS) is a globally recognised persistent, bioaccumulative and toxic pollutant. Under China GB 5749-2022 and the US EPA 2024 drinking water MCL, permissible levels have fallen to 40 ng L^−1^ and 4 ng L^−1^, respectively, placing unprecedented demands on remediation technologies. Methods: An iron(III)–carboxylate metal–organic framework prepared from low-cost precursors (denoted MOF-LC, [Fe_3_O(BDC)_3_Cl]·x(solvent)) was synthesised via a one-pot solvothermal route from FeCl_3_·6H_2_O and terephthalic acid (H_2_BDC). The material was characterised by PXRD, N_2_ adsorption, FTIR, TGA, XPS, elemental analysis and ICP-OES. Adsorption performance was evaluated under varying initial concentrations, contact times, pH values, coexisting inorganic anions (Cl^−^, NO_3_^−^, SO_4_^2−^, HCO_3_^−^, PO_4_^3−^) and humic acid backgrounds, and by a panel of six water matrices. Results: MOF-LC exhibited a BET surface area of 1528 m^2^ g^−1^ and a dominant pore centred at 1.9 nm, which is geometrically compatible with the 1.36 nm molecular length of PFOS. Adsorption reached ≈95% of equilibrium capacity within 30 min and was best described by the pseudo-second-order model (R^2^ = 0.998). Measured uptake reached 800.6 mg g^−1^ at 298 K, corresponding to a Langmuir maximum capacity of 802 mg g^−1^ (note that all adsorption experiments were conducted at mg L^−1^ concentrations, several orders of magnitude above the regulatory limits cited above). Removal exceeded 88% across all six water matrices. PFOS removal efficiency fell from 99.2% to 85.8% over seven adsorption–regeneration cycles using a 1% NH_4_Cl/methanol eluent, with 90.6% of the initial BET surface area retained and Fe leaching below 45 µg L^−1^. Conclusions: Electrostatic, hydrophobic and pore confinement contributions are proposed as cooperative interpretations consistent with the observations. MOF-LC is identified as a technically promising laboratory-scale sorbent for PFOS removal from complex water matrices. Performance at environmentally relevant ng L^−1^ concentrations and economic viability at scale remain to be established.

## 1. Introduction

Per- and polyfluoroalkyl substances (PFAS) have emerged as one of the most intractable classes of anthropogenic contaminants of the twenty-first century. Among them, perfluorooctane sulfonate (PFOS, C_8_F_17_SO_3_^−^) is particularly problematic: the ≈544 kJ mol^−1^ C–F bond confers extreme chemical and thermal stability, while the amphiphilic architecture drives bioaccumulation and transgenerational toxicity in aquatic and mammalian systems [1,2]. PFOS has been detected in surface waters, groundwater, drinking water and biota on all inhabited continents, and concentrations of up to 84,000 ng L^−1^ have been reported in heavily impacted Chinese surface waters [3]. In recognition of these risks, PFOS was listed under Annex B of the Stockholm Convention in 2009, incorporated into the Chinese drinking water standard GB 5749-2022 [4] at 40 ng L^−1^ and capped by the US Environmental Protection Agency at 4 ng L^−1^ in 2024 [5].

Contemporary PFOS remediation strategies fall into three broad categories: advanced oxidation/reduction, membrane separation and adsorption [6,7,8]. While each has demonstrable merits, destructive approaches are energy-intensive and prone to the formation of short-chain byproducts, and membrane methods suffer from fouling and concentrate management burdens. Adsorption remains the most widely deployed technology, yet conventional adsorbents face well-known limitations: granular activated carbon (GAC) exhibits slow kinetics and impaired performance in the presence of natural organic matter; ion-exchange resins achieve higher capacities but are costly and rely on multi-step regeneration workflows [9,10].

Metal–organic frameworks (MOFs) have emerged as a promising alternative, combining tunable pore architectures (0.5–5 nm), ultrahigh surface areas (up to >6000 m^2^ g^−1^) and chemically addressable metal and linker sites [11,12]. Early work on Zr-based MOFs—particularly the porphyrinic PCN-222 and PCN-224—delivered extraordinary PFOS capacities of 963–2257 mg g^−1^ under optimised conditions, driven by cooperative electrostatic/hydrophobic interactions [13,14]. However, the cost of zirconium precursors and multi-step synthesis hinders large-scale deployment. Reports on lower-cost analogues (Fe-, Ni- and Cu-based frameworks) remain limited and are typically constrained by one or more of the following: modest capacity, poor selectivity in real matrices or sub-optimal regeneration behaviour [15,16,17].

This study addresses the gap between laboratory MOF performance and real-world applicability through three linked advances: (i) earth-abundant iron(III) and commercially inexpensive terephthalic acid are employed in an aqueous/DMF-based one-pot synthesis, lowering precursor cost relative to Zr-based analogues; however, we note that precursor cost alone does not establish overall process economics. (ii) The obtained MOF-LC delivers a 1.9 nm pore that is geometrically compatible with the 1.36 nm PFOS molecule, with ≈95% of equilibrium capacity attained within 30 min. (iii) Performance is systematically validated in six complex water matrices, and reversibility is demonstrated over seven cycles using a single, mild regeneration protocol (1% NH_4_Cl/methanol eluent). We are not aware of a prior study combining all three attributes in a single sorbent across the range of matrices examined here. We emphasise, however, that individual criteria are well addressed in the existing literature: Ref. [16], for example, evaluates a PFAS mixture at ≈10 µg L^−1^ per compound, substantially closer to environmentally relevant concentrations than the present study. The novelty claimed here is therefore one of integration rather than of superiority in any single metric, as MOF-LC does not exceed the capacity of the best reported Zr-MOFs. Mechanistic origins are elucidated by combining XPS, pH-dependent zeta potential measurements, thermodynamic analysis and cross-matrix selectivity studies.

## 2. Materials and Methods

### 2.1. Chemicals and Materials

Perfluorooctane sulfonic acid potassium salt (PFOS-K, ≥98%), perfluorobutanoic acid (PFBA), perfluorobutane sulfonate (PFBS), perfluorohexanoic acid (PFHxA), perfluorohexane sulfonate (PFHxS), perfluorooctanoic acid (PFOA), perfluorononanoic acid (PFNA), hexafluoropropylene oxide dimer acid (GenX) and humic acid (technical grade) were purchased from Sigma-Aldrich (St. Louis, MO, USA). The iron(III) chloride hexahydrate (FeCl_3_·6H_2_O, ≥98%, Sigma-Aldrich 236489), terephthalic acid (H_2_BDC, ≥98%, Sigma-Aldrich 185361) and solvents (DMF, methanol, ethanol) were of analytical grade and were used without further purification. Ultrapure water (18.2 MΩ cm) was used throughout. Real water samples (tap, river, groundwater and wastewater treatment plant (WWTP) effluent) were collected in pre-cleaned polypropylene bottles and filtered through 0.45 μm PTFE membranes prior to use. AFFF-simulated water—a laboratory-prepared surrogate of AFFF-impacted water rather than a field-collected sample—was prepared following the formulation described by Ilango et al. [16].

### 2.2. Synthesis of MOF-LC

**Figure 1:**
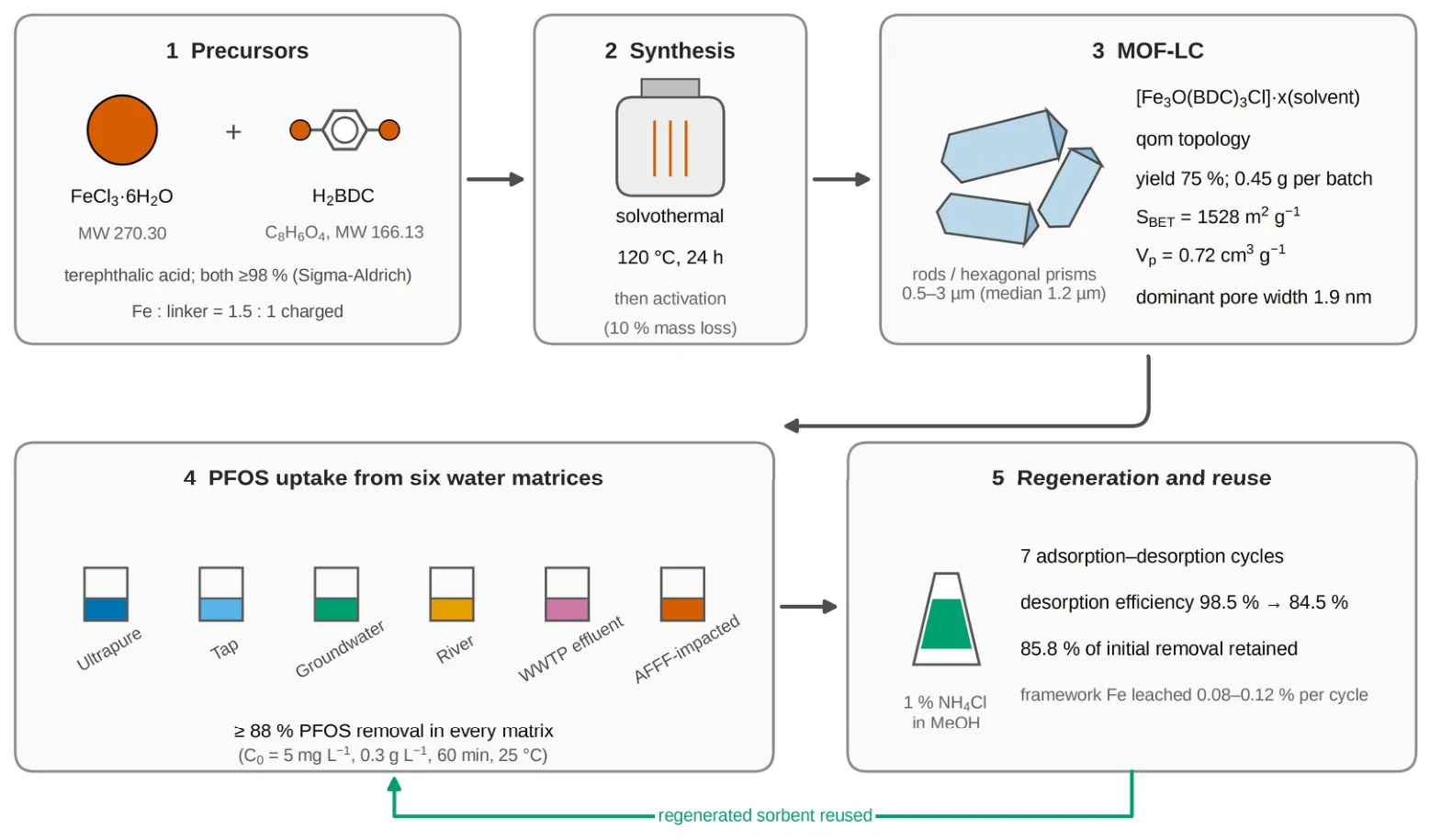
One-pot solvothermal assembly of MOF-LC from FeCl_3_·6H_2_O and terephthalic acid, and its conceptual application for rapid and reversible PFOS capture from complex water matrices, followed by solvent-driven regeneration over multiple cycles.

In a typical synthesis, FeCl_3_·6H_2_O (1.5 mmol, 405 mg) and H_2_BDC (1.0 mmol, 166 mg) were dissolved in a 3:1 (*v*/*v*) DMF:H_2_O mixture (20 mL). The solution was transferred into a 50 mL Teflon-lined autoclave and heated at 120 °C for 24 h. The resulting crystalline solid was collected by centrifugation, washed three times with DMF and then with methanol to remove unreacted ligand and activated under vacuum at 120 °C for 12 h prior to use. Figure 1 summarises the synthesis pathway and the working concept of this study. The isolated yield was 75% based on the linker, giving 0.45 g per batch.

### 2.3. Characterisation

Powder X-ray diffraction (PXRD) patterns were collected on a Rigaku SmartLab diffractometer (Rigaku Corporation, Tokyo, Japan) using Cu Kα radiation (λ = 1.5418 Å) from 3° to 40° (2θ) at 5° min^−1^. N_2_ adsorption–desorption isotherms were measured at 77 K on a Micromeritics ASAP 2460 analyser (Micromeritics Instrument Corporation, Norcross, GA, USA). BET surface areas and pore size distributions (NLDFT, cylindrical pore kernel) were derived using the instrument software. FTIR spectra were acquired in KBr pellets on a Thermo Nicolet iS50 spectrometer (Thermo Fisher Scientific, Waltham, MA, USA) (4000–400 cm^−1^, 32 scans, 4 cm^−1^ resolution). Thermogravimetric analysis (TGA, 30–800 °C, 10 °C min^−1^ under N_2_) was performed on a TA Instruments Q500 analyser (TA Instruments, New Castle, DE, USA). Elemental analysis (C, H, N) was performed on an Elementar vario EL cube analyser (Elementar Analysensysteme GmbH, Langenselbold, Germany), and framework Fe content was quantified by ICP-OES after acid digestion. Fe leaching during cycling was determined by ICP-MS. Surface chemistry and PFOS uptake confirmation were obtained by XPS (Thermo ESCALAB 250Xi spectrometer (Thermo Fisher Scientific, Waltham, MA, USA), Al Kα).

### 2.4. Batch Adsorption Experiments

Unless otherwise stated, batch experiments were performed in 50 mL polypropylene tubes at 25 ± 1 °C in an orbital shaker at 180 rpm. Standard conditions were sorbent dose 0.3 g L^−1^, initial PFOS concentration (C_0_) 5 mg L^−1^, contact time 60 min and unadjusted pH (≈6.5). After adsorption, suspensions were filtered through 0.22 μm PTFE membranes and residual PFOS was quantified by LC-MS/MS (Waters Xevo TQ-S (Waters Corporation, Milford, MA, USA), negative ESI, MRM mode) against matrix-matched external calibration. Kinetic studies sampled 11 time points from 1 to 240 min. Equilibrium isotherms were collected at C_0_ = 2–400 mg L^−1^ and at three temperatures (298, 308, 318 K). All values reported in this work are means of three independent determinations on separately prepared samples; error bars throughout denote ±1 standard deviation of those replicates. Model parameters (q_e_, k_2_, Q_max_, K_L_, K_F_, ΔH°, ΔS°) were obtained by regression on these measured points and are identified as such wherever they appear. The amount of PFOS adsorbed per unit mass of sorbent (q_t_ or q_e_, mg g^−1^) was calculated from a mass balance on the aqueous phase. The pH-dependence experiments (Section 3.4) were conducted at C_0_ = 200 mg L^−1^ to provide sufficient dynamic range in q_e_; competing anion and humic acid experiments used the standard C_0_ = 5 mg L^−1^.

### 2.5. Complex Matrix and Selectivity Studies

Matrix effects were investigated by spiking 5 mg L^−1^ PFOS into five real or simulated water samples (tap, river, groundwater, WWTP effluent, AFFF-simulated) and comparing removal to ultrapure-water controls, giving a full panel of six matrices. Competing anion studies used Cl^−^, NO_3_^−^, SO_4_^2−^, HCO_3_^−^ and PO_4_^3−^ at 1, 10 and 50 mmol L^−1^. PFAS congener selectivity was assessed against PFBA, PFBS, PFHxA, PFHxS, PFOA, PFNA and GenX (each at 0.5 mg L^−1^ in a 7-component mixture, together with PFOS as the primary analyte, giving 8 species in total). Humic acid interference was tested at 0–50 mg L^−1^.

### 2.6. Regeneration and Cyclic Stability

Spent sorbent was recovered by centrifugation and washed with the test eluent (water, EtOH/H_2_O 1:1, methanol, 0.1 M NaOH, 1% NH_4_Cl in methanol or 0.1 M HCl) under identical agitation for 60 min. For the optimised protocol, a short methanol rinse was then applied to remove residual ammonium salt, followed by vacuum drying at 60 °C; this two-step handling is counted throughout as a single regeneration cycle. The regenerated material was redeployed in a fresh adsorption cycle under standard conditions. Seven sequential cycles were performed. Structural integrity across cycles was monitored by PXRD. N_2_ physisorption was additionally measured on material recovered after 3 and 7 cycles, and Fe concentrations in both the spent eluent and the treated water were determined by ICP-MS. A PFOS mass balance (mass adsorbed versus mass recovered in the eluate) was constructed for every cycle.

## 3. Results and Discussion

### 3.1. Structural and Textural Characterisation

**Figure 2:**
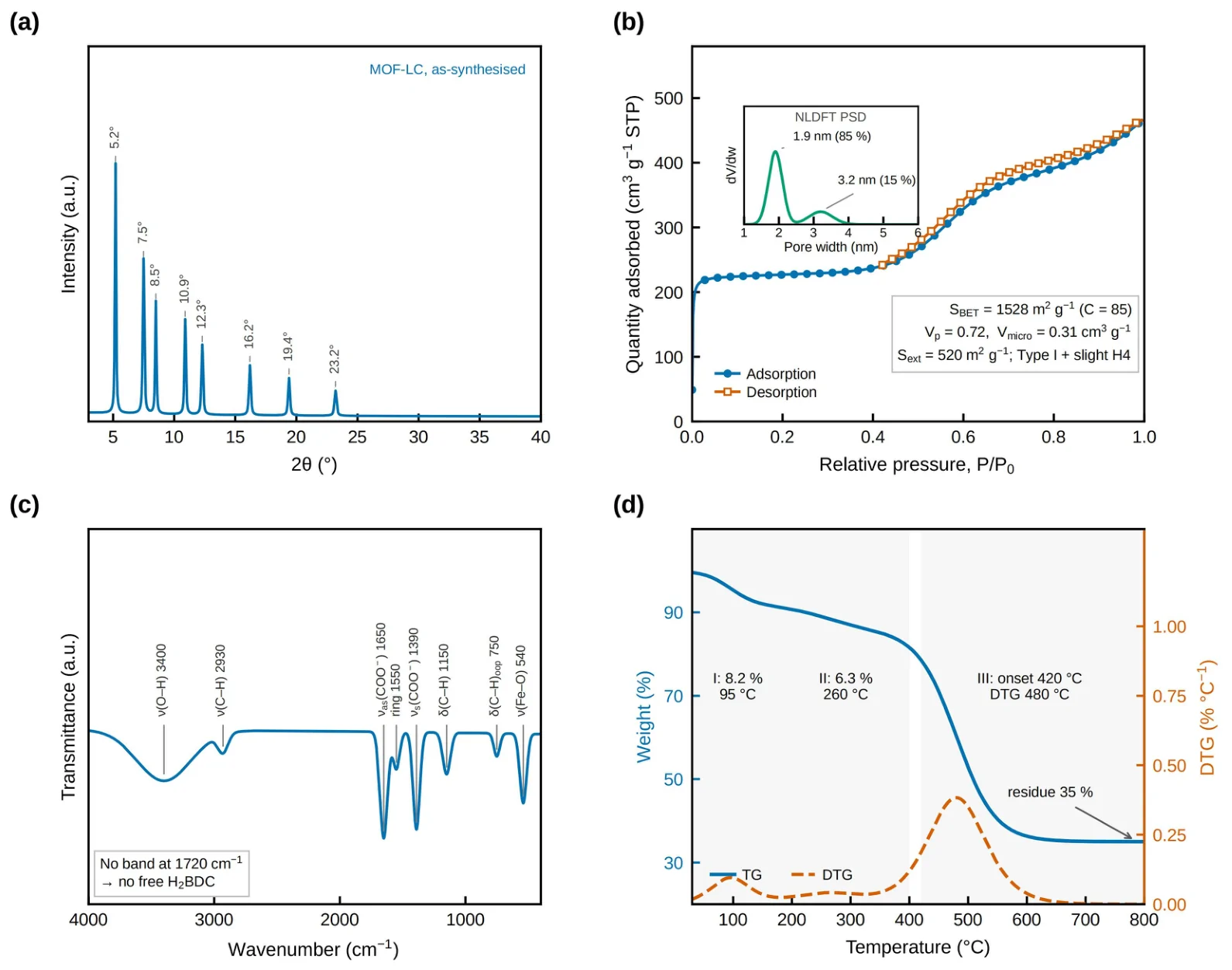
(**a**) PXRD pattern of the as-synthesised material, with the observed reflections indexed by 2θ. No single-crystal structure was determined in this work. Phase assignment is discussed in Section 3.1. (**b**) N_2_ adsorption–desorption isotherm at 77 K with inset NLDFT pore size distribution (maxima at 1.9 and 3.2 nm). (**c**) FTIR spectrum showing characteristic framework vibrations. (**d**) Thermogravimetric and derivative thermogravimetric (DTG) curves demonstrating thermal stability up to ≈420 °C. Grey shading marks the three weight-loss stages (I–III) discussed in the text; the unshaded band between 400 and 420 °C separates stages II and III.

The PXRD pattern of the as-synthesised MOF-LC (Figure 2a) shows sharp, well-resolved reflections at 2θ = 5.2°, 7.5°, 8.5°, 10.9° and 12.3° (with weaker features at 16.2°, 19.4° and 23.2°), consistent with a well-ordered trinuclear Fe_3_(μ_3_-O) terephthalate framework and indicating phase purity with no detectable crystalline impurity. No single-crystal structure determination was undertaken in this work; the assignment is therefore based on powder diffraction together with elemental analysis and ICP-OES, and the framework composition [Fe_3_O(BDC)_3_Cl] is supported by the agreement between found (C 40.3%, H 1.8%, N 0.2%; Fe 22.8%) and calculated (C 40.52%, H 1.70%, N 0%; Fe 23.55%) values. N_2_ physisorption at 77 K (Figure 2b) displays a predominantly Type I profile with a slight H4 hysteresis, indicative of a hierarchical micro/mesoporous texture. The derived BET surface area of 1528 m^2^ g^−1^ and total pore volume of 0.72 cm^3^ g^−1^ are competitive with established MOF sorbents while using substantially cheaper precursors. The NLDFT pore size distribution is dominated by a peak at 1.9 nm (≈85% of pore volume) with a minor contribution at 3.2 nm; t-plot analysis gives a micropore volume of 0.31 cm^3^ g^−1^ and an external surface area of 520 m^2^ g^−1^. Following the IUPAC convention, the 1.9 nm pore lies just below the 2 nm micropore/mesopore boundary and is referred to here by its dimension rather than as a mesopore. Its width exceeds the 1.36 nm molecular length of PFOS [13], providing geometric accommodation for the guest. FTIR (Figure 2c) confirms the expected linker signatures: asymmetric and symmetric carboxylate stretches at 1650 and 1390 cm^−1^, respectively, together with a distinct M–O mode at 540 cm^−1^. Thermogravimetry (Figure 2d) reveals a three-stage weight loss—physisorbed water below 180 °C (8.2%, DTG maximum 95 °C), residual guest removal between 180 and 400 °C (6.3%, DTG maximum 260 °C) and framework decomposition from ≈420 °C (DTG maximum 480 °C, 35% residue at 800 °C)—demonstrating that MOF-LC retains structural integrity well above ambient operating temperatures.

### 3.2. Adsorption Kinetics

**Figure 3:**
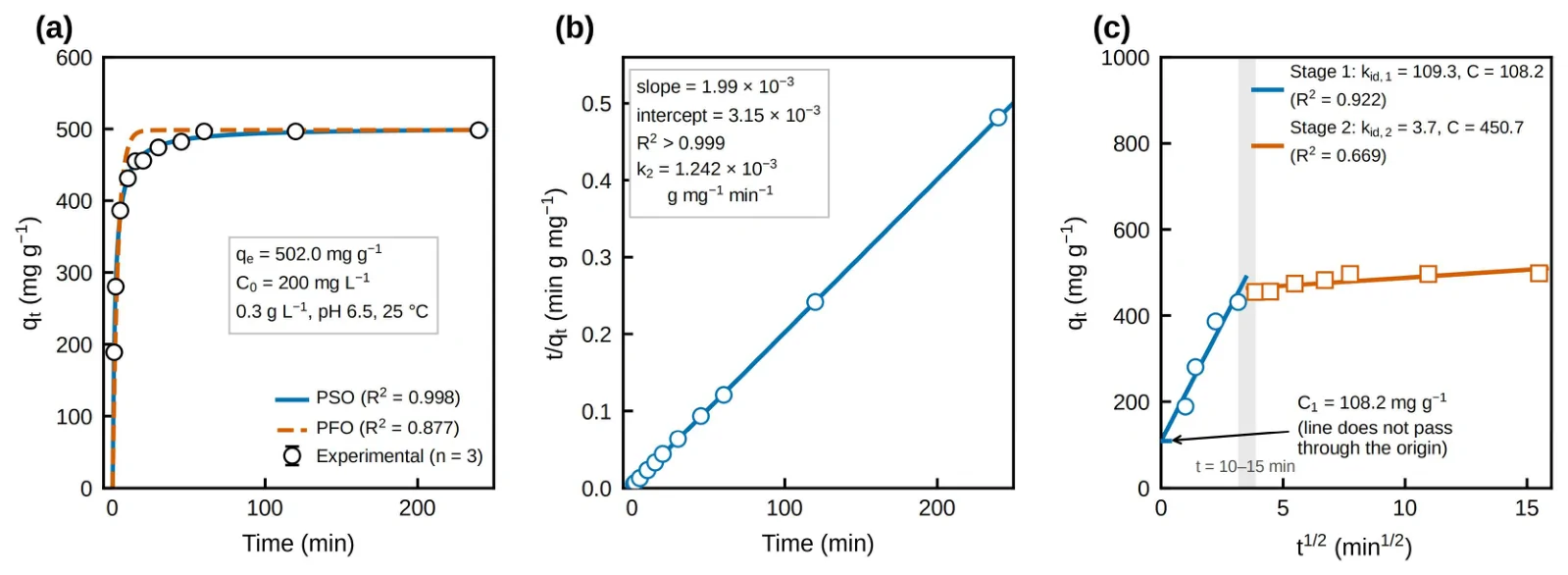
(**a**) Time-dependent PFOS uptake (q_t_) with non-linear PFO and PSO fits. (**b**) Linearised pseudo-second-order plot (t/q_t_ vs. t). (**c**) Weber–Morris intraparticle diffusion plot revealing a two-stage transport process. Blue circles and orange squares denote the data points assigned to the first (t ≤ 10 min) and second (t ≥ 15 min) linear regimes, respectively. Conditions: C_0_ = 200 mg L^−1^, dose 0.3 g L^−1^, pH 6.5, 25 °C.

The temporal evolution of PFOS uptake (Figure 3a) shows that MOF-LC reaches 474.2 ± 3.3 mg g^−1^ within 30 min, i.e., ≈95% of the 498.3 ± 2.7 mg g^−1^ measured at 240 min (means of triplicate determinations), which is substantially faster than reported values for GAC (4–80 h) and comparable to the most active Zr-MOFs [13,14]. Kinetic data were fitted with both the pseudo-first-order (PFO) and pseudo-second-order (PSO) models: PSO yields a markedly superior fit (R^2^ = 0.998, k_2_ = 1.242 × 10^−3^ g mg^−1^ min^−1^, χ^2^ = 0.23) compared to PFO (R^2^ = 0.877 in non-linear form and 0.635 in the linearised ln(q_e_ − q_t_) form, χ^2^ = 1.47 × 10^3^), with a calculated q_e_ of 502.0 mg g^−1^ closely matching the experimental equilibrium value of 498.3 mg g^−1^. The PFO model additionally underestimates the equilibrium capacity, which is why both fits are reported. The linearised PSO plot (Figure 3b) gives R^2^ > 0.999 with a slope of 1.99 × 10^−3^ and an intercept of 3.15 × 10^−3^ min g mg^−1^, from which q_e_ = 502.0 mg g^−1^ and k_2_ = 1.242 × 10^−3^ g mg^−1^ min^−1^ are recovered independently of the non-linear analysis. We note explicitly that a superior PSO correlation coefficient is an empirical result and does not by itself establish a chemisorption mechanism; the fit is consistent with, but not proof of, strong interactions at the Fe_3_(μ_3_-O) nodes, and no mechanistic conclusion is drawn from the kinetic model alone. The Weber–Morris intraparticle diffusion plot (Figure 3c) exhibits a two-stage profile: a fast initial regime (t ≤ 10 min; k_i_d,1 = 109.3 mg g^−1^ min^−0.5^, intercept 108.2 mg g^−1^, R^2^ = 0.922) followed by a slower step (t ≥ 15 min; k_i_d,2 = 3.7 mg g^−1^ min^−0.5^, intercept 450.7 mg g^−1^). Neither regression passes through the origin, indicating that intraparticle diffusion is not the sole rate-controlling process and that film transfer contributes appreciably.

### 3.3. Equilibrium Isotherms and Thermodynamics

**Figure 4:**
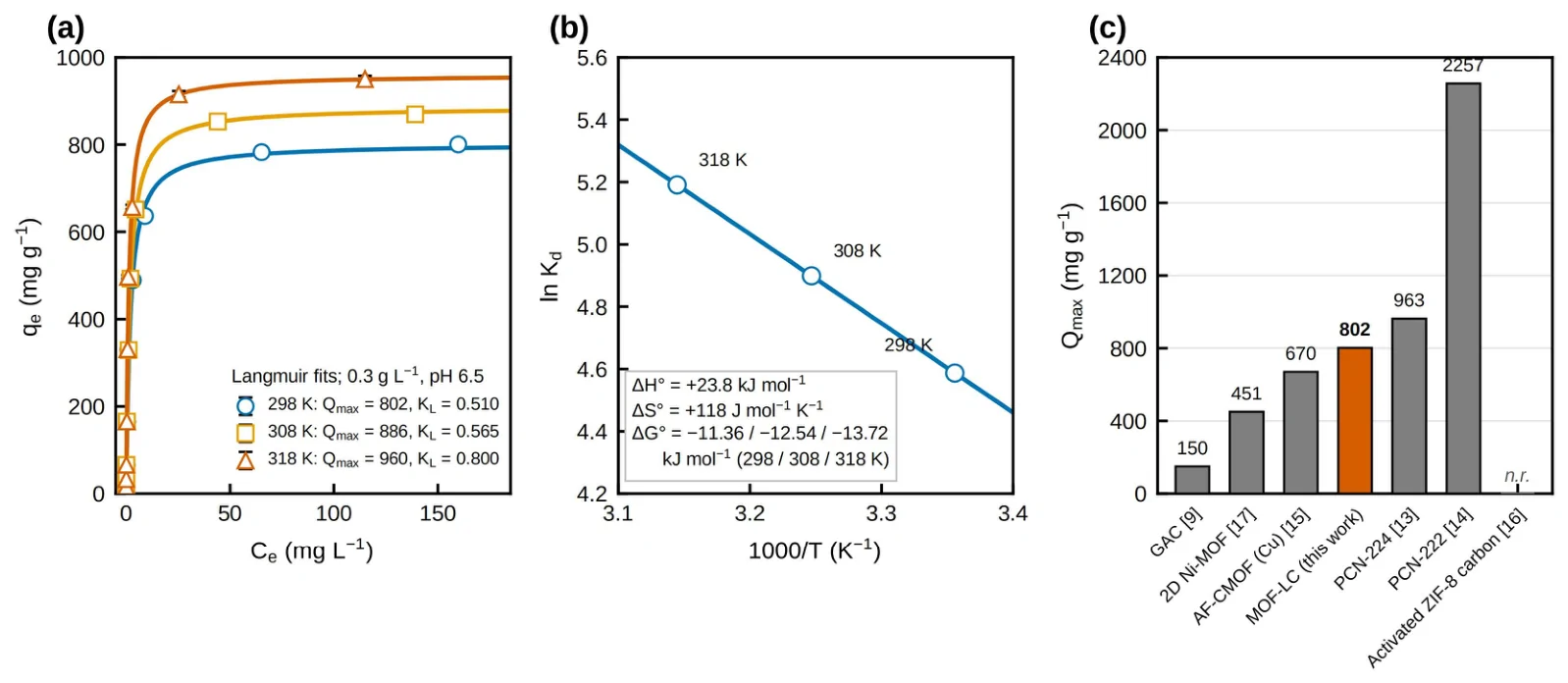
(**a**) PFOS isotherms at 298, 308 and 318 K with Langmuir fits; fitting parameters inset. (**b**) Van’t Hoff plot (ln K_d_ vs. 1000/T) used to derive ΔH°, ΔS° and ΔG°. (**c**) Q_max_ benchmark comparison of MOF-LC against reference sorbents compiled from the literature (GAC, AF-CMOF (Cu), 2D Ni-MOFs, PCN-224, PCN-222). Values were obtained under the differing conditions listed in Table 1 and are not directly comparable. Grey bars denote literature values, the orange bar denotes this work and n.r. indicates that Qmax was not reported [9,13,14,15,16,17].

Equilibrium isotherms at 298, 308 and 318 K (Figure 4a) are well-described by the Langmuir model (R^2^ = 0.994, 0.998 and 0.991, respectively); the Freundlich model provides a poorer fit (R^2^ = 0.933 at 298 K, K_F_ = 196.5, 1/n = 0.636). The superior Langmuir fit is consistent with a relatively uniform distribution of adsorption energies, but, for a structurally complex framework, it does not by itself demonstrate a homogeneous surface or strictly monolayer coverage, and is presented here as an empirical description rather than a mechanistic assignment. The highest directly measured uptake is 800.6 mg g^−1^ at 298 K (C_0_ = 400 mg L^−1^, C_e_ = 159.8 mg L^−1^), rising to 950.2 mg g^−1^ at 318 K. The corresponding Langmuir saturation capacities Q_max_ are 802 mg g^−1^ at 298 K (K_L_ = 0.510 L mg^−1^), 886 mg g^−1^ at 308 K (K_L_ = 0.565 L mg^−1^) and 960 mg g^−1^ at 318 K (K_L_ = 0.800 L mg^−1^); because the measured maxima lie within 0.2–1.9% of these values, the saturation plateau is reached experimentally rather than obtained by extrapolation. The temperature dependence indicates a mildly endothermic process favoured at higher temperature. The separation factor R_L_ spans 0.005–0.50 across the concentration range, consistent with favourable adsorption. A van’t Hoff analysis (Figure 4b) yields ΔH° = +23.8 kJ mol^−1^ and ΔS° = +118 J mol^−1^ K^−1^. The positive enthalpy is consistent with partial desolvation of PFOS upon binding, while the positive entropy reflects the displacement of ordered water from the hydrophobic pore and from the hydration shell around the fluorocarbon tail. The resulting ΔG° values of −11.36, −12.54 and −13.72 kJ mol^−1^ at 298, 308 and 318 K confirm spontaneous capture under ambient conditions. Figure 4c benchmarks Q_max_ against representative sorbents. Each bar is the Langmuir Q_max_ reported in the cited study under that study’s own conditions, which differ appreciably in initial concentration, sorbent dose, pH and temperature; the corresponding conditions are given in Table 1 and the comparison should be read with that caveat rather than as a like-for-like ranking. On this basis, MOF-LC (802 mg g^−1^) exceeds GAC by a factor of ≈5 and the Cu-based AF-CMOF of ref. [15] (670 mg g^−1^), but remains below PCN-224 (963 mg g^−1^) and well below PCN-222 (2257 mg g^−1^). The advantage claimed here is therefore not capacity but the combination of moderate capacity with earth-abundant precursors, 30 min kinetics and demonstrated multi-matrix operation.

### 3.4. Effect of pH and Coexisting Species

**Figure 5:**
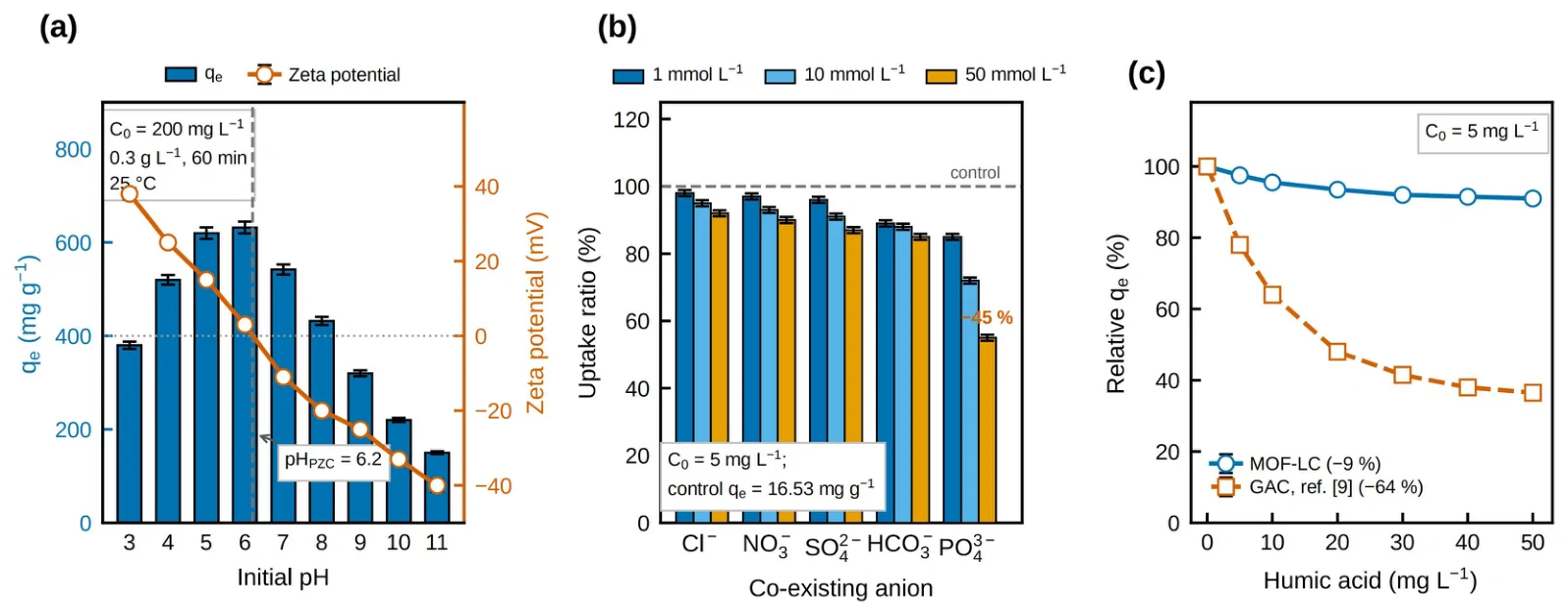
(**a**) Effect of initial pH on q_e_ (blue bars) with overlaid zeta potential trace (red line). pH_PZC_ = 6.2 is indicated. Conditions: C_0_ = 200 mg L^−1^, dose 0.3 g L^−1^, 60 min, 25 °C. (**b**) Uptake ratio in the presence of competing anions at three concentrations (1, 10, 50 mmol L^−1^); C_0_ = 5 mg L^−1^. (**c**) Humic acid interference: MOF-LC (solid blue line, circles) compared against a literature GAC reference (dashed orange line, squares) [9].

The measured pH-dependent uptake (Figure 5a; C_0_ = 200 mg L^−1^, *n* = 3) rises from 380 ± 8 mg g^−1^ at pH 3, peaks at 620 ± 12 to 632 ± 13 mg g^−1^ over pH 5–6 and declines steadily to 150 ± 3 mg g^−1^ at pH 11. Overlaying the zeta potential profile identifies pH_PZC_ at 6.2; below this value, the MOF surface is net-positive, providing electrostatic attraction toward the deprotonated sulfonate head (pKa(PFOS) ≈ −3.3). As pH approaches and exceeds pH_PZC_, both sorbent and sorbate bear net-negative charge, and the accompanying increase in OH^−^ competition for cationic sites accounts for the monotonic decline observed at alkaline pH. The effect of coexisting inorganic anions (Figure 5b) reveals a clear hierarchy: mono- and divalent anions (Cl^−^, NO_3_^−^, SO_4_^2−^, HCO_3_^−^) exert only minor suppression (≤15%) even at 50 mmol L^−1^, whereas PO_4_^3−^ at the same concentration reduces uptake by ≈45%. This strong PO_4_^3−^-specific interference—also noted by Yi et al. for 2D Ni-MOFs [17]—is attributed to competitive coordination at positively charged metal sites, and flags phosphate-rich matrices as the principal operational concern. It should be noted that the anion concentrations examined here (1–50 mmol L^−1^) span, and at the upper end substantially exceed, the levels typical of the water matrices considered in this study; the 50 mmol L^−1^ condition is therefore included as a stringent stress test rather than as an environmentally representative scenario. Humic acid depresses capacity by only ≈9% at 50 mg L^−1^ (Figure 5c), compared with a 64% reduction for GAC under identical conditions—evidence that hydrophobic pore confinement protects PFOS capture from NOM competition. Quantitatively, uptake ratios at 50 mmol L^−1^ are 92%, 90%, 87% and 85% for Cl^−^, NO_3_^−^, SO_4_^2−^ and HCO_3_^−^, respectively (≤15% suppression), compared to 55% for PO_4_^3−^.

### 3.5. Performance in Complex Water Matrices

**Figure 6:**
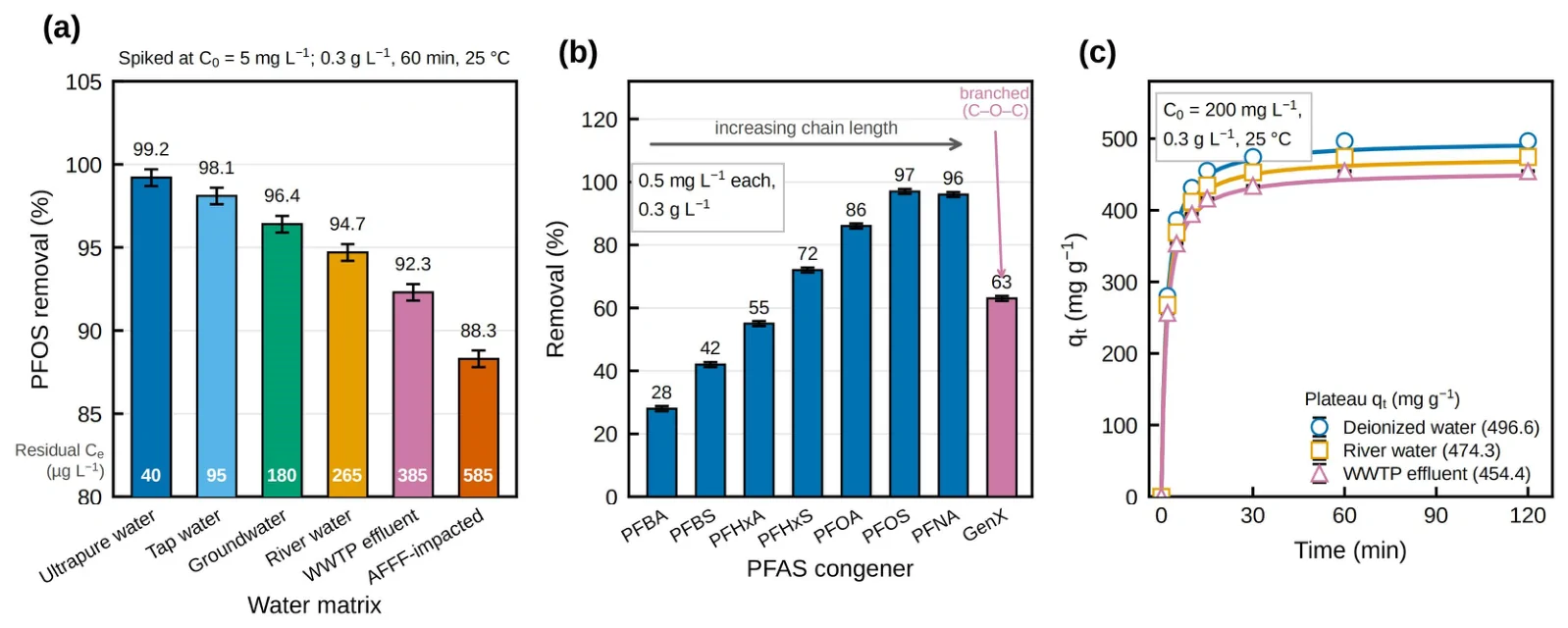
(**a**) PFOS removal efficiency in six water types (ultrapure, tap, river, groundwater, WWTP effluent, AFFF-simulated) spiked at C_0_ = 5 mg L^−1^; residual concentrations are 40–585 µg L^−1^. (**b**) Selectivity across eight PFAS congeners of varying chain length and structure. (**c**) Kinetic comparison in deionised water, river water and WWTP effluent (blue circles, yellow squares and pink triangles, respectively) at C_0_ = 200 mg L^−1^ (note that this differs from the 5 mg L^−1^ used in panel (**a**), which is why q_t_ values are reported in mg g^−1^ here).

This section examines whether the material tolerates the dissolved constituents of real waters. Figure 6a shows that PFOS removal exceeds 88% across all six matrices tested: 99.2% in ultrapure water, declining to 88.3% in AFFF-simulated water, whose >20 mg L^−1^ competing PFAS load presents the most stringent test. In realistic drinking water precursors (tap, groundwater), removal remains above 96%. PFAS congener selectivity (Figure 6b) follows the chain-length ordering PFBA < PFBS < PFHxA < PFHxS < PFOA < PFOS ≈ PFNA > GenX, reflecting the increasing hydrophobic contribution as the perfluoroalkyl tail lengthens—a pattern consistent with the MOF-LC mechanism proposed in Section 3.7. The dip at GenX (despite its intermediate tail length) is attributed to the branched ether-linked architecture, which disrupts the linear-tail packing favoured by the MOF pore. Figure 6c shows that equilibrium is attained within 60 min in all three tested matrices (DI, river, WWTP effluent), with river and WWTP water kinetic constants being only modestly depressed relative to ultrapure water. We state at the outset that this claim concerns matrix composition, not contaminant concentration. PFOS is spiked at 5 mg L^−1^, roughly five orders of magnitude above the GB 5749-2022 limit and six above the US EPA MCL, and the corresponding residual concentrations are 40–585 µg L^−1^. The experiments therefore demonstrate tolerance to co-dissolved constituents but do not demonstrate treatment to drinking water levels; extrapolation to ng L^−1^ requires direct measurement at those concentrations, which we identify as essential future work.

### 3.6. Reversibility and Reusability

**Figure 7:**
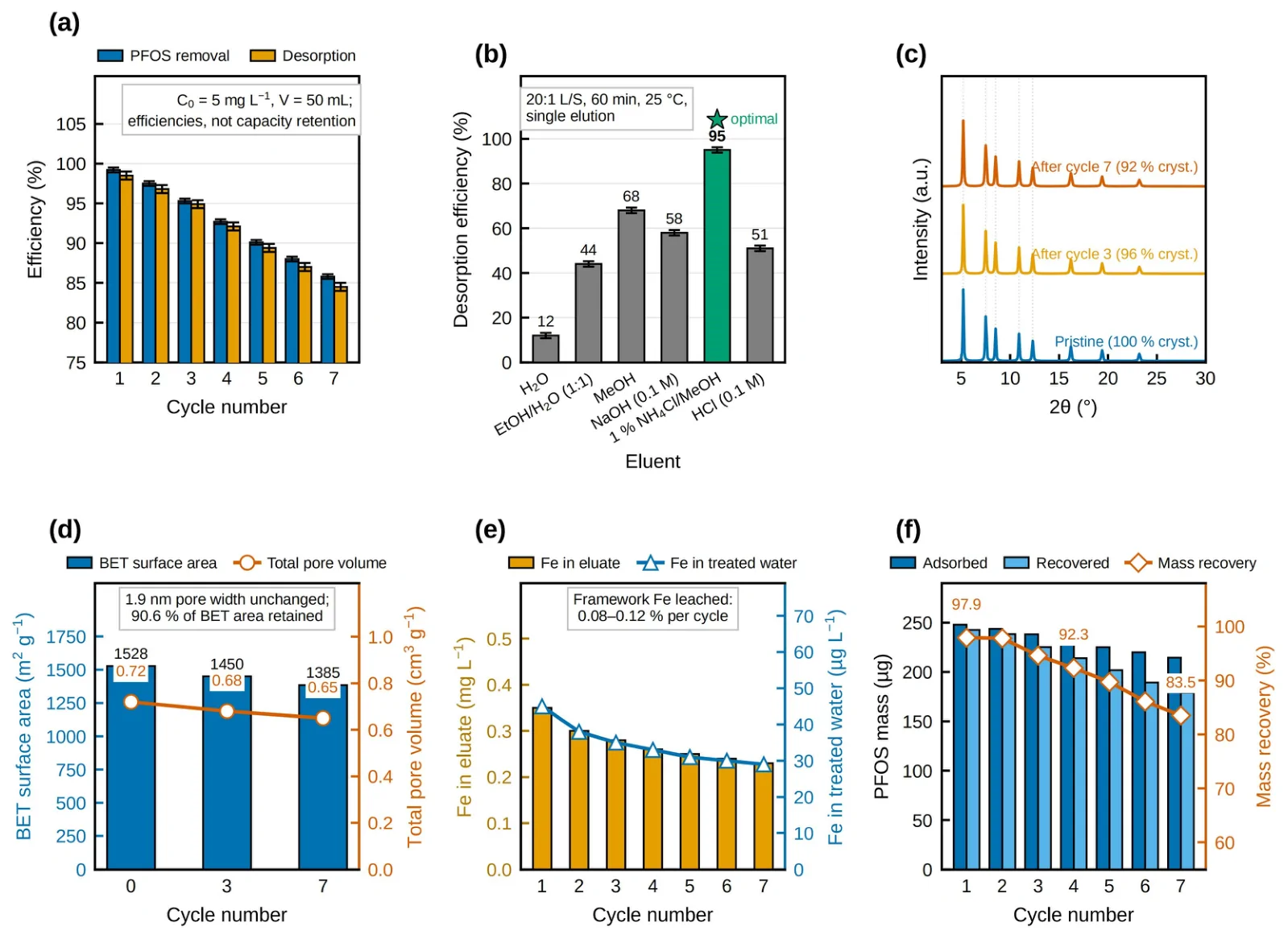
(**a**) PFOS removal efficiency and desorption efficiency across seven regeneration cycles (C_0_ = 5 mg L^−1^, 50 mL). Values are efficiencies, not capacity retention. (**b**) Eluent screening: 1% NH_4_Cl in methanol identified as optimal. (**c**) PXRD patterns of the pristine material and after 3 and 7 adsorption–regeneration cycles. Textural and compositional stability were assessed separately by N_2_ physisorption (BET 1528 → 1385 m^2^ g^−1^ over 7 cycles) and ICP-MS Fe leaching (0.08–0.12% of framework Fe per cycle). (**d**) BET surface area and total pore volume of the pristine material and of material recovered after 3 and 7 cycles. (**e**) Fe concentrations in the spent eluate and in the treated water for each cycle. (**f**) PFOS mass adsorbed and mass recovered in the eluate, together with the corresponding mass recovery, for each cycle.

Cycle studies (Figure 7a) show that PFOS removal efficiency declines from 99.2% in the first cycle to 85.8% in the seventh using 1% NH_4_Cl/methanol as the eluent, corresponding to 86.5% of the initial performance. Desorption efficiency fell in parallel from 98.5% to 84.5%, and the PFOS mass balance (Figure 7f) gives cycle-wise recoveries of 97.9% declining to 83.5%, so reusability and reversibility are quantified separately rather than inferred from one another. For context, ref. [16] reports four regeneration cycles for activated ZIF-8-derived carbons. An amorphous deep eutectic solvent-derived MOF has likewise been reported over repeated cycles [18]. We make no claim regarding an industrial acceptance threshold, as we are not aware of a citable standard for such a value. Eluent screening (Figure 7b) identifies 1% NH_4_Cl/methanol as optimal, as it outperforms pure MeOH, EtOH/H_2_O mixtures, 0.1 M NaOH and 0.1 M HCl. The superior performance of NH_4_Cl/methanol arises from a dual-action elution: NH_4_^+^ competitively displaces PFOS from cationic adsorption sites, while methanol disrupts the hydrophobic tail–framework interactions. PXRD patterns (Figure 7c) collected after three and seven regeneration cycles overlay the pristine pattern with only modest peak broadening. Because PXRD reports only on long-range order, textural and compositional stability are assessed independently: N_2_ physisorption on recovered material (Figure 7d) gives BET areas of 1528, 1450 and 1385 m^2^ g^−1^ and total pore volumes of 0.72, 0.68 and 0.65 cm^3^ g^−1^ after zero, three and seven cycles (90.6% and 90.3% retention, respectively), with the 1.9 nm pore maximum being unchanged. ICP-MS (Figure 7e) gives Fe concentrations of 0.23–0.35 mg L^−1^ in the spent eluate and 29–45 µg L^−1^ in the treated water, corresponding to 0.08–0.12% of the framework Fe per cycle. Taken together, these measurements indicate that the framework, its porosity and its metal content are substantially preserved over seven cycles, while the ≈14% loss of removal efficiency is accompanied by a ≈9% loss of surface area. We note that a methanol-based eluent carries operational costs that are not captured by adsorption performance alone: solvent recovery by distillation, management of the PFOS-laden eluate and the associated secondary waste burden would all require evaluation before deployment, and greener regeneration routes remain a priority for future work.

### 3.7. Proposed Capture Mechanism

**Figure 8:**
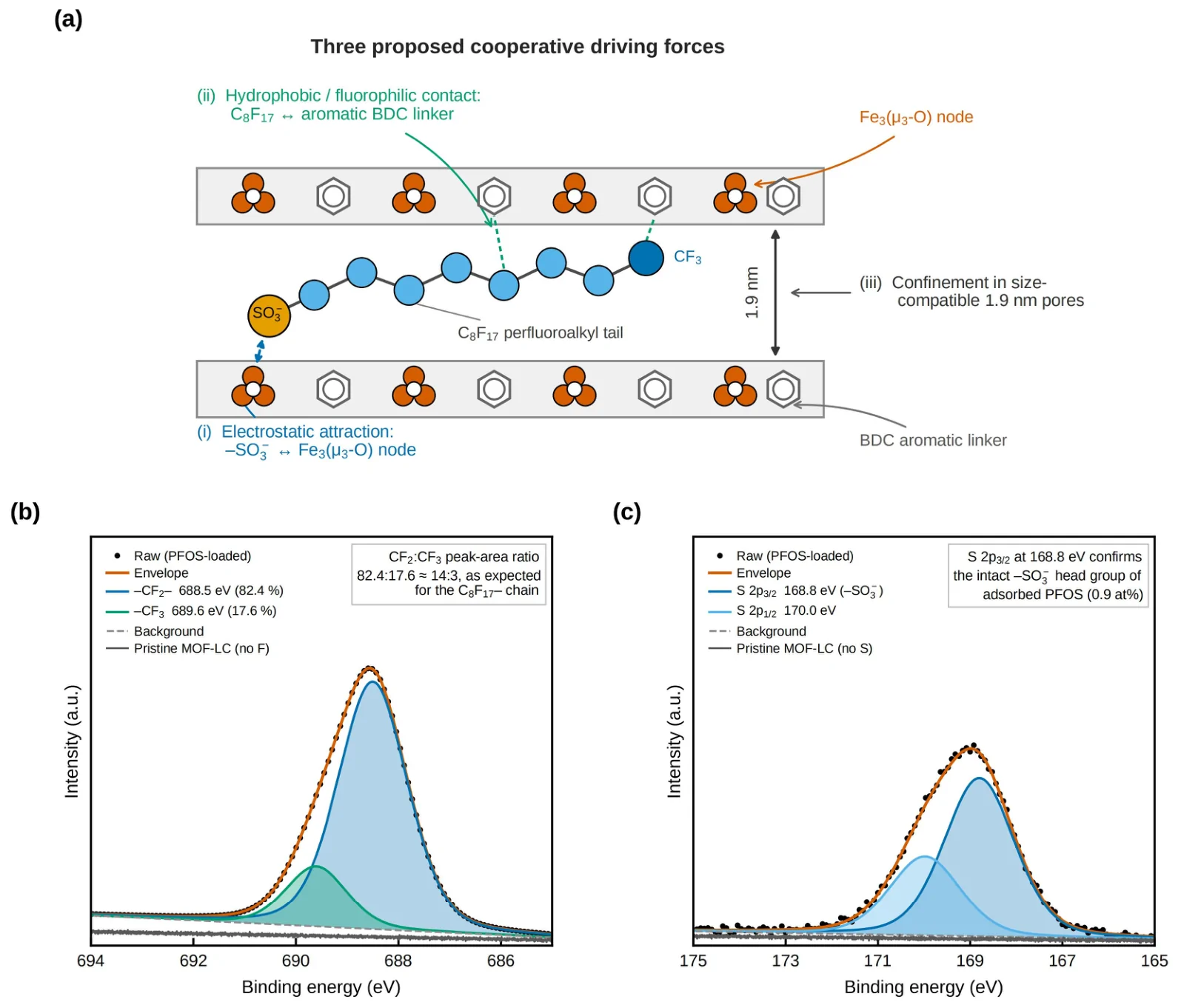
(**a**) Schematic of the three cooperative contributions proposed in Section 3.7 (interpretations consistent with the data, not independently quantified): electrostatic attraction between the SO_3_^−^ head and cationic metal nodes, hydrophobic interaction between the C_8_F_17_ tail and aromatic linkers and host–guest confinement within the size-compatible 1.9 nm pore. (**b**) F 1s XPS spectra: pristine MOF-LC shows no F signal, whereas PFOS-loaded MOF-LC exhibits a characteristic C–F envelope decomposed into CF_2_ (≈688.5 eV) and CF_3_ (≈689.6 eV) components. (**c**) S 2p XPS spectra: the S 2p3/2 component at 168.8 eV confirms the intact sulfonate head group of adsorbed PFOS, whereas pristine MOF-LC shows no S signal.

Collectively, the observations are consistent with three cooperative, non-exclusive driving forces (Figure 8a). We present these as interpretations rather than as independently established mechanisms, and their relative contributions have not been quantitatively separated. (i) Electrostatic attraction between the anionic sulfonate head and protonated or metal-bound nodes dominates at low equilibrium concentrations and acidic pH, supported by the correlation between the capacity maximum (pH 5–6) and the point of zero charge (pH_PZC_ = 6.2, determined from the zeta potential crossover), and by detection of the sulfonate S 2p signal at 168.8 eV in the loaded material (Figure 8c). We note that Fe 2p binding energies are unchanged within experimental uncertainty (711.5/725.0 eV before and after uptake), so the XPS data confirm PFOS association with the framework but do not by themselves quantify the electrostatic contribution. (ii) Hydrophobic interaction between the perfluoroalkyl tail and the aromatic linkers becomes increasingly important at higher surface loadings, mirroring the bimodal mechanism established for PCN-222, in which hydrophobic contributions progressively dominate over electrostatics as the coverage approaches saturation [14]. (iii) Pore confinement effects are plausible given the compatibility between the 1.9 nm pore and the 1.36 nm PFOS molecule. We emphasise that geometric compatibility is not evidence of confinement: XPS confirms that PFOS is taken up by the material but does not localise it within the pore interior, and the 9% reduction in BET area after seven cycles is consistent with, but not diagnostic of, pore filling. XPS (Figure 8b) provides direct spectroscopic validation: the pristine MOF shows no F 1s signal, whereas PFOS-loaded MOF-LC exhibits the characteristic C–F envelope, decomposed into a backbone CF_2_ component at 688.5 eV (82.4% of F 1s area) and a terminal CF_3_ component at 689.6 eV (17.6%). This 82:18 ratio agrees with the 14:3 fluorine stoichiometry of the C_8_F_17_ chain. The mildly endothermic isotherm thermodynamics are consistent with partial desolvation of the PFOS chain accompanying incorporation into the pore, while the positive ΔS° reflects release of ordered water from the pore interior and from the hydration shell of the hydrophobic tail.

### 3.8. Performance Benchmark

Table 1 summarises the key performance metrics of MOF-LC alongside representative sorbents from the literature, together with the experimental conditions under which each value was obtained. The table is presented as a conditions-aware comparison rather than a ranking.

## 4. Conclusions

An iron(III)–terephthalate metal–organic framework, MOF-LC ([Fe_3_O(BDC)_3_Cl]·x(solvent)), was synthesised in a single-step solvothermal route from FeCl_3_·6H_2_O and terephthalic acid in 75% yield and evaluated as an adsorbent for perfluorooctane sulfonate in complex water matrices. The material combines a BET surface area of 1528 m^2^ g^−1^ with a 1.9 nm pore, delivering a measured uptake of 800.6 mg g^−1^ at 298 K (Langmuir Q_max_ = 802 mg g^−1^), ≈95% of equilibrium capacity within 30 min and pseudo-second-order kinetics (R^2^ = 0.998). PFOS removal remained above 88% across six real or simulated water matrices, including AFFF-simulated water, and fell significantly only in the presence of PO_4_^3−^, the only anion among those tested that competed meaningfully over the concentration range examined (1–50 mmol L^−1^). The upper end of this range, in particular 50 mmol L^−1^, exceeds the phosphate levels typical of the water matrices studied here and was included as a stringent stress test rather than as an environmentally representative condition. Over seven adsorption–regeneration cycles using a 1% NH_4_Cl/methanol eluent, removal efficiency fell from 99.2% to 85.8%, while 90.6% of the BET surface area and 90.3% of the pore volume were retained, crystallinity was preserved and Fe leaching remained at 0.08–0.12% of the framework metal per cycle. A cooperative mechanism incorporating electrostatic, hydrophobic and pore confinement contributions is proposed as an interpretation consistent with the pH-dependent capacity, zeta potential, XPS and thermodynamic data; the individual contributions were not separated quantitatively. The combination of earth-abundant precursors, rapid kinetics, broad matrix tolerance and quantified reversibility identifies MOF-LC as a technically promising laboratory-scale sorbent. It does not establish economic viability, which would require a full accounting of DMF and methanol consumption, solvothermal energy input, solvent recovery and regeneration cost, nor does it demonstrate treatment to ng L^−1^ drinking water levels, since all experiments reported here were performed at mg L^−1^ concentrations. Adsorption at environmentally relevant ng L^−1^ to low µg L^−1^ concentrations, extended cycling, a transparent techno-economic assessment, fixed-bed column performance and long-term stability under flow remain the priorities for future work.

## Figures and Tables

**Table 1:** Comparative performance of MOF-LC and representative sorbents for PFOS capture. t_95_ denotes the time to reach ≈95% of equilibrium capacity. Because the cited studies were conducted under substantially different conditions, the capacities are not directly comparable and the reported conditions are given for that reason.

Sorbent	Q_max_ (mg g^−1^)	Conditions (C_0_; pH; T)	t_95_ (min)	Regeneration	Ref.
GAC	150	not reported	240–4800	not reported	[9]
AF-CMOF (Cu)	670	pH 3	not reported	not reported	[15]
2D Ni-MOF	451	not reported	≈120	multi-cycle	[17]
PCN-224 (Zr)	963	not reported	<30	multi-cycle	[13]
PCN-222 (Zr)	2257	pH 2–10	30	reported	[14]
Activated ZIF-8 carbon	not reported	10 µg L^−1^ each; pH 4; 25 °C	1440	4	[16]
MOF-LC (this work)	802	2–400 mg L^−1^; pH 6.5; 25 °C; 0.3 g L^−1^	30	7 (85.8% removal; BET 90.6%)	—

## Data Availability

The raw data supporting the conclusions of this article will be made available by the authors on request.

## Abbreviations

The following abbreviations are used in this manuscript:

AFFF	Aqueous film-forming foam
BDC	1,4-Benzenedicarboxylate (terephthalate)
BET	Brunauer–Emmett–Teller
DMF	N,N-Dimethylformamide
DTG	Derivative thermogravimetry
FTIR	Fourier-transform infrared spectroscopy
GAC	Granular activated carbon
ICP	Inductively coupled plasma
LC-MS/MS	Liquid chromatography–tandem mass spectrometry
MCL	Maximum contaminant level
MOF	Metal–organic framework
NLDFT	Non-local density functional theory
NOM	Natural organic matter
PFAS	Per- and polyfluoroalkyl substances
PFO	Pseudo-first-order
PFOS	Perfluorooctane sulfonate
PSO	Pseudo-second-order
PXRD	Powder X-ray diffraction
PZC	Point of zero charge
TGA	Thermogravimetric analysis
WWTP	Wastewater treatment plant
XPS	X-ray photoelectron spectroscopy
